# First clinical experience with belzutifan in von Hippel–Lindau disease associated CNS hemangioblastoma

**DOI:** 10.2217/cns-2022-0008

**Published:** 2022-07-12

**Authors:** Andrew Dhawan, David M Peereboom, Glen HJ Stevens

**Affiliations:** 1 Neurological Institute, Cleveland Clinic, Cleveland, OH 44195, USA; 2 Taussig Cancer Institute, Cleveland Clinic, Cleveland, OH 44195, USA

**Keywords:** belzutifan, hemangioblastoma, VHL, von Hippel–Lindau

## Abstract

We present two cases of von Hippel–Lindau (VHL) disease-associated hemangioblastomas in the CNS treated with the newly approved HIF-2α inhibitor, belzutifan. The first case is a 31-year-old female with confirmed pathogenic germline *VHL* mutation who presented with multiple hemangioblastomas. The patient was started on belzutifan, and a brisk reduction in perilesional edema was observed after 2 months of treatment. The second patient is a 30-year-old male with familial VHL disease. Imaging revealed multiple cerebellar hemangioblastomas, and follow-up imaging after three cycles of belzutifan revealed a reduction in perilesional edema. Both patients tolerated belzutifan well, with only anemia and fatigue. We highlight our initial experience and early imaging findings associated with belzutifan in VHL disease-associated CNS hemangioblastomas.

Von Hippel–Lindau (VHL) disease is an autosomal dominant inherited tumor susceptibility syndrome due to germline mutation in the *VHL* gene [1]. The disease is characterized by the development of malignancies in multiple organs, most commonly clear cell renal cell carcinoma, pancreatic neuroendocrine tumors, endolymphatic sac tumors, epididymal cystadenomas and CNS hemangioblastomas [1]. CNS hemangioblastomas are present in 60–80% of patients with VHL disease, and of these 45% are in the cerebellum, 36% are in the spinal cord and the remainder are in brainstem, cauda equina, supratentorium, pituitary stalk, optic nerve and spinal nerve roots [2]. The majority are asymptomatic with major manifestations being headaches, hiccoughs, nausea, vomiting, paresthesias and vertigo, owed primarily to their mass effect and posterior fossa location [2,3].

The *VHL* gene encodes a protein involved in the E3 ubiquitin ligase complex, stabilized by interactions with the elongin B and elongin C proteins [4]. Through these stabilizing interactions, in normoxia, this E3 ubiquitin ligase complex is itself resistant to proteasomal degradation and acts to target hydroxylated hypoxia-inducible factors (HIFs) for degradation [5]. In conditions of hypoxia, HIFs are not hydroxylated and are not targeted for degradation, thereby allowing them to fulfill essential roles as transcription factors activating multiple cellular programs such as apoptosis, vascular proliferation, cellular growth and anaerobic respiration enabling the cell to potentially survive hypoxia [6]. Thus, when the *VHL* gene harbors a mutation with protein dysfunction preventing its binding to elongin B and elongin C, the E3 ubiquitin ligase complex is destabilized and in conditions of normoxia, HIFs are no longer targeted for degradation. This results in constitutive activation of HIFs, causing an aggressive cellular phenotype that can develop into malignancy [6].

HIFs themselves comprise a set of multiple heterodimeric complexes with three distinct α subunits (HIF-1α, -2α and -3α), recently shown to have unique functions [6]. Experimentally, it has been shown that HIF-2α confers an aggressive growth and vascular proliferative phenotype that may drive malignancy in patients with VHL disease [7]. This finding led to the development of a targeted inhibitor of HIF-2α, belzutifan (MK 6482, PT-2977), now approved for use in patients with VHL disease-associated renal cell carcinomas, pancreatic neuroendocrine tumors and CNS hemangioblastomas not requiring immediate surgery [8–10]. Efficacy of belzutifan in patients with renal carcinoma and germline *VHL* alterations was established by the clinical trial NCT 03401788. Of the patients in this trial, 39% had CNS hemangioblastomas and 63% of patients with CNS hemangioblastomas experienced a radiographic response, although the imaging features of this response are not noted in the trial results [10]. The most common adverse effects reported with belzutifan have been anemia, fatigue, headache, impairment of renal function, dizziness and nausea [10].

In this manuscript, we present two cases of VHL disease with a strong early response of CNS lesions to the targeted agent belzutifan, representing our early experience with this drug. We present the imaging findings in each of these patients to highlight the degree of responses that we have seen. The first is the case of a 31-year-old female with a confirmed pathogenic germline variant in the *VHL* gene, with multiple intracranial lesions and a cervical spinal cord lesion all compatible with hemangioblastoma. She was treated with belzutifan after surgical resection for a cerebellar lesion. In follow-up imaging, the patient had a significant reduction in the amount of perilesional edema, tumor diameter and contrast enhancement. Likewise, the second case we present is that of a 30-year-old male with genetically confirmed VHL disease with multiple posterior fossa hemangioblastomas, with progressive disease after resection and stereotactic radiosurgery (SRS), which was complicated by radiation necrosis. Due to the progression of his cerebellar hemangioblastomas, he was started on belzutifan, which was tolerated well, and a reduction in tumor diameter and perilesional edema was seen on follow-up imaging after three cycles.

## Case 1: history & examination

The patient is a 31-year-old Caucasian right-handed female with genetically confirmed VHL disease (heterozygous deletion of exon 2 in *VHL* gene, sequenced on the Illumina platform to at least 50× depth, NC_000003.12:g.(?_10146504)_(10146646_?)del), who presented with recurrent headaches 3 years after the resection of multiple cerebellar hemangioblastomas. Her initial presentation was over 5 years prior to the neurology clinic with left occipital headaches and amenorrhea for 18 months. MRI brain with and without intravenous contrast done at this time was revealing of multiple presumed cerebellar hemangioblastomas. MRI of the cervical spine revealed a high cervical lesion at this time as well. She was also found to have a right retinal hemangioblastoma and left renal lesion. On abdominal imaging, she was found to have asymptomatic pancreatic cysts. She underwent resection of three posterior fossa hemangioblastomas, with histopathological confirmation of the diagnosis. Her postoperative course was complicated by infection and pseudomeningocele formation over the site of resection, ultimately treated by aspiration and antibiotics. She remained clinically stable, though because of prolonged steroid doses, she suffered avascular necrosis of the hips and shoulders.

About 3 years after the initial presentation, she represented to the neurology clinic with headaches, and MRI brain identified a cystic lesion in the left cerebellopontine angle, concerning for interval development of hemangioblastoma. Due to lesion progression on an interval scan 1 month later, the patient underwent resection of this mass. Unexpectedly, on histopathology, examination of the mass revealed a WHO grade 1 pilocytic astrocytoma with a Ki-67 index of 3–4%. She recovered well postoperatively without requiring any steroids. The examination was unremarkable except for unsteadiness with Romberg’s test with sway to the left and an inability to perform tandem gait. The remainder of the cranial nerve, sensory and motor examinations were reported as normal.

### Belzutifan initiation & follow-up

**Figure 1.**
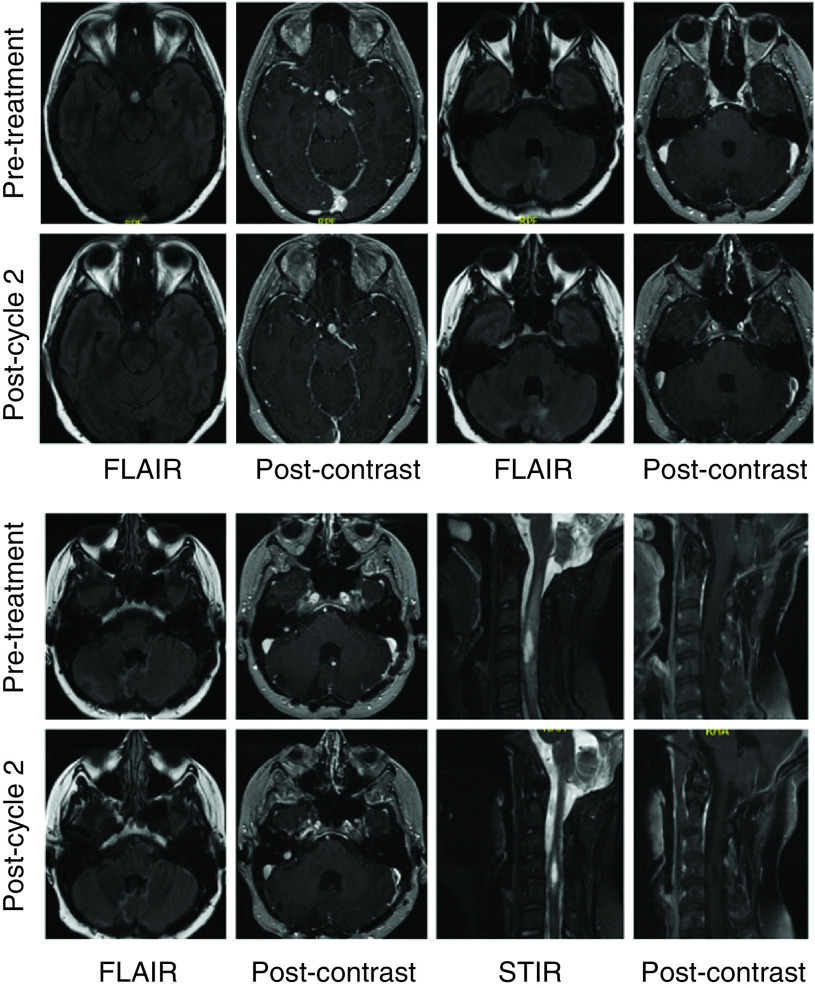
Pre- and post-treatment MRI brain and cervical spine for patient 1. Interval MRI of the brain and cervical spine performed with and without intravenous gadolinium contrast reveals a decrease in the degree of enhancement of intracranial lesions (pituitary area, multiple posterior fossa lesions, cervical lesion) and reduced perilesional FLAIR and STIR signal intensity. T1 postcontrast images of the cervical spine are fat suppressed. FLAIR: Fluid-attenuated inversion recovery; STIR: Short-TI inversion recovery.

Due to the presence of the left renal lesion, right retinal hemangioblastoma and unresectable intracranial and spinal hemangioblastomas with progressive nodularity at the site of the resected pilocytic astrocytoma, the patient was started on belzutifan 120 mg given orally once daily. Follow-up imaging after two cycles showed significant improvement in perilesional edema around intracranial and spinal hemangioblastomas (Figure 1). Her right cerebellar lesion had gone from 10 to 8.5 mm in diameter, and her cerebellar nodule had gone from 3 to 2 mm in diameter. There was a 10 mm suprasellar mass that was 9 mm in diameter on repeat imaging. Her right-sided cervical cord lesion had gone from 18 mm × 7 mm × 10 mm with 4 mm enhancing nodule to 17 mm × 9 mm × 6 mm with 3 mm enhancing nodule. On examination, the patient demonstrated good comprehension with a ‘brain fog’ sensation but had normal cranial nerve, motor and sensory examinations. Her gait was mildly antalgic, but no dysmetria or dysdiadochokinesia was observed. Over the course of her therapy, hemoglobin and creatinine were monitored. She experienced a decrease in hemoglobin from 12.9 to 11.0 g/dl after cycle 1, and a further decrease to 9.8 g/dl after cycle 2. Her creatinine remained stable. There were no other toxicities noted.

## Case 2: history & examination

The patient is a 30-year-old left-handed Caucasian male with genetically confirmed VHL disease, who was diagnosed at age 10. He presented initially with left eye vision loss due to retinal hemangioblastoma and is blind in the left eye secondary to surgical management. He also was found at the time of diagnosis to have known brain and spinal hemangioblastomas. With regards to the systemic manifestations of VHL disease, he has renal, pancreatic and epididymal cysts that are being followed radiographically. His medications include pancreatic enzyme replacement after he developed weight loss secondary to diarrhea and was found to have pancreatic insufficiency.

**Figure 2.**
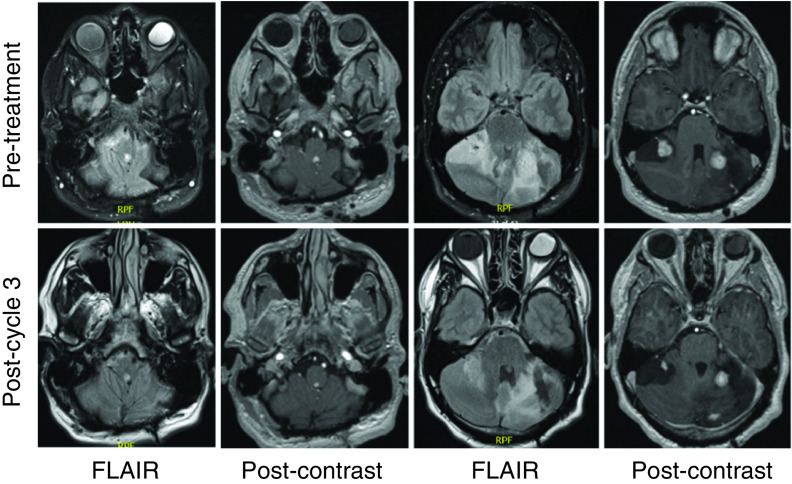
Pre- and post-treatment MRI imaging for patient 2. The time interval between scans was 4 months, and the patient had received three cycles of belzutifan. Panels show fourth ventricular mass and multiple cerebellar hemangioblastomas, with interval reduction in perilesional edema and reduced contrast enhancement. FLAIR: Fluid-attenuated inversion recovery.

He underwent surgery first at age 15 years for a cerebellar hemangioblastoma and was followed radiographically until age 24 years. At age 24 years, he developed posterior neck tightness, headaches, and paresthesias in the feet and hands. Imaging revealed progressive disease, and he underwent resection of a second deep left cerebellar mass, a superficial cerebellar mass, a dorsal fourth ventricular mass, and had a C1 laminectomy for Chiari decompression. He required wound washout two weeks after surgery but otherwise recovered well. At age 25 years, he had worsening headaches and neck pain, with imaging showing again progressive disease in the posterior fossa. He underwent SRS to a lesion in the left cerebellum, which was complicated by radiation necrosis post-treatment. At 9 months post-treatment, he was started on bevacizumab for radiation necrosis, of which he completed three of four planned cycles, and stopped due to a superficial skin infection. He continued to be monitored radiographically, and while the examination was without any neurologic deficits, at age 29 years, he was found to have progressive disease in three cerebellar hemangioblastomas on follow-up imaging (Figure 2).

### Belzutifan initiation & follow-up

For his progressive cerebellar hemangioblastomas, he was started on belzutifan at a dose of 120 mg given orally once daily. At the time of the first follow-up, he had completed three cycles of belzutifan and underwent repeat MRI imaging of the brain, which showed interval improvement in perilesional edema for the posterior fossa lesions. His dominant left lateral cerebellar lesion had decreased in diameter from 2.7 to 1.9 cm, and his dominant right cerebellar hemisphere lesion had decreased in diameter from 2.5 to 1.7 cm. A right superior cerebellar hemispheric lesion decreased from 9 to 3 mm in enhancing diameter, and a dorsal medulla lesion decreased from 7 to 4 mm in diameter. His hemoglobin has dropped from 15.3 pre-treatment to 10.4 g/dl post-treatment cycle 3, but his creatinine is stable. He experienced mild fatigue and dizziness since starting belzutifan but has otherwise remained asymptomatic. There are no new neurological deficits or findings on physical examination.

## Discussion

In this manuscript, we present the first reported imaging findings in two patients with VHL disease treated with the novel targeted agent belzutifan. This report highlights the rapid resolution of perilesional edema associated with intracranial and spinal hemangioblastomas after starting belzutifan. Both patients we report tolerated the drug well, without major systemic toxicities, though both did experience anemia with mild–moderate fatigue. Prior to the approval of belzutifan, there was no targeted therapy for the management of CNS tumors in patients with VHL disease, and in these patients, SRS and repeat resection were relatively contraindicated due to extensive prior surgical history and known radiation and steroid-related complications.

Efficacy of belzutifan was established by NCT 03401788, which was an open-label clinical trial for patients with renal cell carcinoma and a *VHL* germline alteration [9,10]. Among the 61 patients enrolled in this trial, all had at least one solid lesion in the kidney and 24 of these patients had CNS hemangioblastomas [10]. These patients did not receive any prior systemic chemotherapy. The spatial distribution of hemangioblastomas in the CNS among these patients is not provided. Efficacy results in the drug label state that 63% of patients (95% CI: 41–81%) with hemangioblastoma had a response. Of these 15 patients, one had a complete response and 14 others had a partial response. Median response duration was not reached in the trial and 11 of 15 of those who had a partial or complete response had a duration of at least 12 months. Criteria used to assess response were RECIST 1.1 criteria, but not explicitly stated for hemangioblastomas.

## Conclusion

Our report is limited as it represents only two cases of early radiologic and clinical response to belzutifan initiation. However, this report serves to highlight the brisk initial responses we saw in terms of imaging characteristics that may be expected when starting belzutifan. Thus, while both patients tolerated belzutifan well initially and have shown brisk responses, a longer study duration will establish the durability of response and tolerability of therapy.

**Executive summary**

- Von Hippel–Lindau (VHL) disease is an autosomal dominant inherited tumor susceptibility syndrome, with multisystem manifestations including CNS hemangioblastomas, clear cell renal carcinoma and endolymphatic sac tumors.
- VHL disease occurs due to germline mutations in the *VHL* gene.
- The *VHL* gene codes for a protein that degrades hypoxia-inducible factors (HIFs) that enable the cell to survive hypoxia.
- In VHL disease, HIFs are constitutively expressed even in normoxia, resulting in dysregulated cell growth throughout multiple tissues.
- Belzutifan is a novel HIF-2α inhibitor that has been approved for use in CNS hemangioblastomas, clear cell renal carcinoma and pancreatic neuroendocrine tumors.
- While efficacy has been demonstrated in CNS hemangioblastomas, the imaging characteristics for patients undergoing treatment with belzutifan have not been described.
- We describe two patients in our center’s first experience with belzutifan, and show that there was early reduction in CNS hemangioblastoma size, as well as reduction in perilesional edema.
- Both patients tolerated belzutifan well, with no dose-limiting toxicities.
